# Semaglutide in Obesity and Type 2 Diabetes Management: A Systematic Review of Clinical Outcomes

**DOI:** 10.7759/cureus.78555

**Published:** 2025-02-05

**Authors:** Sri Ram Charan Gundapaneni, Rithwik Goud Burri, Rohini Kaku, Aiturgan Mamytova, Poshitha Kondadasula, Tugolbai Tagaev

**Affiliations:** 1 Department of General Medicine, Rangaraya Medical College, Kakinada, IND; 2 Department of General Medicine, Maheshwara Medical College and Hospital, Hyderabad, IND; 3 Department of General Medicine, I.K. Akhunbaev Kyrgyz State Medical Academy, Bishkek, KGZ; 4 Department of General Medicine, A.C. Subba Reddy Government Medical College, Nellore, IND; 5 Department of Hospital Internal Medicine With a Course of Hematology, I.K. Akhunbaev Kyrgyz State Medical Academy, Bishkek, KGZ

**Keywords:** adverse effects, efficacy, glucagon-like peptide-1 receptor agonist, glycemic control, obesity, semaglutide, type 2 diabetes, weight loss

## Abstract

Semaglutide, a glucagon-like peptide-1 receptor agonist, has emerged as a promising pharmacological intervention in obesity management. This systematic review aimed to evaluate the efficacy and safety of semaglutide in promoting weight loss in adults with obesity. A comprehensive literature search was conducted in the PubMed, Scopus, and Web of Science databases, and nine studies met the inclusion criteria. The studies included randomized controlled trials and case-control studies involving participants aged 18-65 years with BMI > 30 kg/m². Semaglutide administered once a week, particularly at a dosage of 2.4 mg, led to a significant mean weight loss of 14.9% compared to 2.4% in the group receiving a placebo (mean difference: -12.4 percentage points; 95% CI: -13.4 to -11.5; P < 0.001). Research has indicated weight reductions between 3 and 15 kg, with greater doses and longer treatment durations, resulting in more substantial decreases. Additionally, semaglutide considerably improved glycemic control, with 57-74% of participants receiving 1.0 mg achieving HbA1c levels below 7.0% (P < 0.0001). The observed improvements in weight and metabolic parameters were consistent across the diverse racial and demographic groups. The most common adverse effects observed were mild-to-moderate gastrointestinal issues, primarily nausea and vomiting. These symptoms were dose-dependent and typically transient, although they caused some participants to discontinue the treatment. Assessment of potential bias using the Cochrane Risk of Bias Tool revealed that most studies had a low overall risk, with some exhibiting moderate risks in areas of performance, detection, and reporting biases. In conclusion, semaglutide is effective as a pharmacological intervention for obesity management, resulting in significant weight loss and improved glycemic control. However, long-term safety data remain scarce, and gastrointestinal side effects may negatively affect patient compliance. Further research is required to optimize dosing strategies, assess cost-effectiveness, and evaluate long-term safety, thus enhancing the clinical utility and accessibility of the drug.

## Introduction and background

The obesity epidemic involves a complex interplay between modifiable and non-modifiable risk factors that affect countries globally at various developmental stages. Modifiable factors include dietary habits, physical inactivity, and environmental factors [1], whereas non-modifiable factors include genetics, age, ethnicity, sex, and physiological changes [1,2]. Studies have emphasized the role of diet, particularly high-calorie food intake and low omega-3 consumption, among the risk factors [3]. Genetic factors contribute significantly to obesity risk, accounting for 40-70%, but their expression is greatly influenced by environmental and behavioral factors [2,4]. Childhood obesity often predicts adult obesity, highlighting the need for early intervention. Addressing this public health issue requires a holistic approach that considers both modifiable lifestyle choices and non-modifiable genetic factors, as well as environmental influences and individual behaviors [1,5,6].

Semaglutide, a glucagon-like peptide-1 (GLP-1) receptor agonist, has been approved for the treatment of type 2 diabetes mellitus (T2DM) in both injectable and oral forms, with the oral form being the first GLP-1 receptor agonist in pill form [7,8]. It enhances insulin release in response to glucose and possesses a prolonged half-life owing to its resistance to dipeptidyl peptidase-4 (DPP-4) breakdown [9]. Semaglutide improves glycemic control and reduces cardiovascular risk factors in patients with T2DM [8,10].

Semaglutide also exhibits promise beyond glycemic control, inducing significant weight loss and receiving FDA approval for obesity treatment [11]. Additionally, it has demonstrated protective effects on the liver in non-alcoholic steatohepatitis and neuroprotective properties in Parkinson’s and Alzheimer’s diseases [11]. Diabetes management outperforms placebo by reducing glycated hemoglobin (HbA1c) levels by >1%, largely owing to a 5% decrease in body weight [12]. Semaglutide yields promising results in the context of weight loss and diabetes control compared to other glucagon-like peptide-1 and dipeptidyl peptidase-4 receptor agonists, but it causes more severe gastrointestinal side effects, including nausea and diarrhea [13].

Semaglutide has therapeutic applications for T2DM, obesity, and other metabolic and neurodegenerative disorders. Its oral form satisfies the demand for non-injectable diabetes treatment, potentially improving patient compliance and quality of life [14], and its efficacy for reducing epicardial adipose tissue thickness and its favorable safety profile further underscore its clinical use [10,15].

Additionally, a recent study demonstrated improved efficacy and safety for treating patients with T2DM and severe obesity using sodium-glucose cotransporter-2 inhibitor therapy [16]. Although semaglutide is proposed to offer neuroprotective and hepatoprotective benefits, especially in conditions such as non-alcoholic steatohepatitis and neurodegenerative disorders, these assertions require confirmation through comprehensive clinical studies [17,18]. Current research has focused on metabolic advantages, leaving a broader impact of semaglutide in managing chronic diseases.

An increasing number of studies and clinical trials investigating semaglutide-based medications have aimed to confirm the effect of the drug on weight loss and its effectiveness as an obesity treatment in adults. This review assessed the effectiveness and safety of semaglutide in treating obesity by examining weight reduction, blood sugar regulation, and side effects across clinical trials. It also addresses research limitations by emphasizing the need for extended safety data, refinement of dosage protocols, and investigation of the efficacy of semaglutide relative to emerging therapeutic options.

## Review

Methods

This systematic review followed the guidelines for Preferred Reporting Items for Systematic Reviews and Meta-Analyses [19].

Search Strategy

A comprehensive search was performed in various scientific databases such as PubMed, Scopus, and Web of Science to identify key studies focused on semaglutide as a potential obesity treatment. The main search terms included "semaglutide and obesity", "semaglutide", and "obesity". Database-specific search strings are given as follows.

PubMed search string: The PubMed search string is given as follows: 

("semaglutide"[Title/Abstract] OR "GLP-1 receptor agonist"[Title/Abstract])

AND ("obesity"[Title/Abstract] OR "weight loss"[Title/Abstract] OR "type 2 diabetes"[Title/Abstract])

AND ("randomized controlled trial"[Publication Type] OR "clinical trial"[Publication Type])

AND (humans[MeSH] AND English[lang])

AND (2018:2024[dp])

Scopus search string: The Scopus search string is given as follows:

TITLE-ABS-KEY(semaglutide OR "GLP-1 receptor agonist")

AND TITLE-ABS-KEY(obesity OR "weight loss" OR "type 2 diabetes")

AND TITLE-ABS-KEY("randomized controlled trial" OR "clinical trial")

AND PUBYEAR > 2017

AND (LIMIT-TO(LANGUAGE, "English"))

Web of Science search string: The Web of Science search string is given as follows:

TS=(semaglutide OR "GLP-1 receptor agonist")

AND TS=(obesity OR "weight loss" OR "type 2 diabetes")

AND TS=("randomized controlled trial" OR "clinical trial")

AND PY=(2018-2024)

AND LA=(English)

Eligibility Criteria

Inclusion criteria: The primary selection criteria included English-language publications between January 2018 and May 2024. Specific criteria encompassed peer-reviewed original research, randomized clinical trials (RCTs), case-control studies, pseudo-experimental studies, adults (aged 18-65 years) with a body mass index (BMI) >30 kg/m², and studies reporting weight loss, glycemic control, or adverse effects. Although restricting research to English-language sources may exclude some relevant studies, this criterion ensures precise data extraction and interpretation. Moreover, many of the most influential studies on obesity and diabetes have been published in English, reducing the likelihood of overlooking crucial findings.

Exclusion criteria: The exclusion criteria comprised case reports, editorials, review articles, studies lacking a placebo or comparator group, in vivo or in vitro studies, and studies where the full text was unavailable.

Although RCTs are considered the gold standard for evaluating pharmacological interventions, case-control and pseudo-experimental studies were included to provide additional insights into real-world effectiveness, treatment adherence, and variations in patient responses beyond the controlled environment of RCTs. Research articles, review articles, and RCTs were examined to assess the relationship between medication administration and weight loss outcomes.

Data Extraction

We examined variables including identification data (title, authors, journal, publication year, and country), methodology (study type and evidence level), number of participants, age, obesity status, post-treatment weight loss, and medication dosage. The contributions of each study were summarized by extracting patient data, standardizing weight loss measurements to kilograms, and evaluating weight reduction according to age and treatment duration. Both authors screened titles and abstracts using Rayyan QCRI, a systematic review management tool that facilitates blinded screening, applied the inclusion and exclusion criteria, removed redundant data, and imported the remaining results into EndNote 20 software.

Titles and abstracts from the search results were initially reviewed for eligibility based on the inclusion and exclusion criteria, followed by a full manuscript review to confirm the necessary components for the study. The identification, selection, eligibility, and inclusion of studies followed the Preferred Reporting Items for Systematic Reviews and Meta-Analyses (PRISMA) criteria [19].

To ensure consistent data extraction, three independent reviewers (N.V., L.V., and M.B.) followed a standardized protocol. Disagreements were resolved through discussion and consultation with a senior reviewer if necessary. A pre-established template ensured uniformity in capturing variables such as study design, sample characteristics, intervention specifics, outcome measures, and statistical significance.

Risk of Bias Assessment

The Cochrane Risk of Bias Tool (RoB 2.0) was used to assess potential bias in the selected studies, focusing on selection, performance, detection, reporting, and overall risk. Two independent reviewers evaluated each study, resolving disagreements through discussion or consultation with a third reviewer. Bias levels were categorized as low risk (green), moderate risk (yellow), or high risk (red). A visual heatmap was used to compare risk levels across all studies, providing a comprehensive overview of the assessment results.

Results

**Figure 1:**
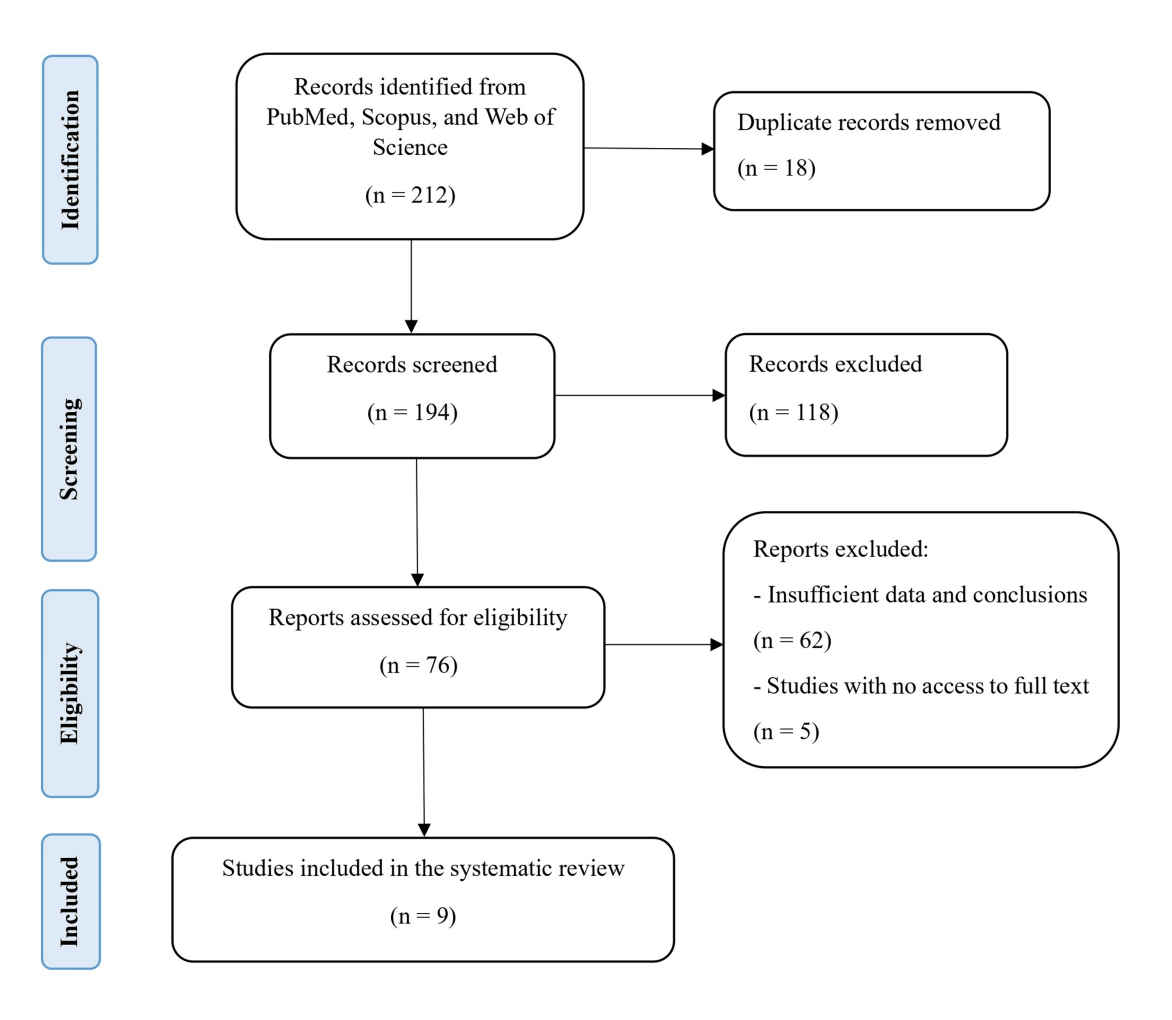
PRISMA flow diagram of literature search and study selection for systematic review PRISMA: Preferred Reporting Items for Systematic Reviews and Meta-Analyses.

The initial search of PubMed, Scopus, and Web of Science databases yielded 212 records. After eliminating 18 duplicates, 194 articles remained. The screening process excluded 118 records that were irrelevant or did not meet the inclusion criteria. Of the 76 reports assessed for eligibility, 62 were excluded due to inadequate data or conclusions, and five were omitted because full texts were unavailable despite attempts to gain access. Ultimately, nine studies met the inclusion criteria and were included in the systematic review [20-28]. Figure 1 depicts the selection process, and Table 1 presents a summary of the key features of the included studies.

**Table 1:** Characteristics of selected studies on the association between semaglutide and its potential use in obesity DFUs, diabetic foot ulcers; HBOT, hyperbaric oxygen therapy; SC, standard care; HbA1c, glycated hemoglobin; T2DM, type 2 diabetes mellitus.

Study details	Sample	Mean age and BMI	Weight reduction	Dosage and time for treatment	Main results
Wilding et al. [20]	Patients with obesity	46 years and 37.8 kg/m^2^	Loss of 15.3 kg	2.4 mg and 68 weeks	Over 68 weeks, the semaglutide group exhibited a 15.3 kg weight change compared to a 2.6 kg change in the placebo group.
Kushner et al. [21]	Patients with obesity	46.5 years and Between 35.7–38.5 kg/m^2^	Patients maintained their weight with a high-calorie diet.	2.4 mg and 68 weeks	Anti-obesity drugs are a treatment option important for people who live with obesity and cannot lose and maintain weight loss or for those who do not comply.
Desouza et al. [22]	All patients with T2DM, 64% of patients have obesity	50 years and 33–34.6 kg/m^2^	Loss of 2.3–4.7 kg and 3.6–6.1 kg	0.5–1 mg and 56 weeks	In patients with T2DM, semaglutide consistently and clinically reduced HbA1c levels and body weight, with similar efficacy and safety across various racial and ethnic groups.
Devries et al. [23]	Patients with obesity	53.7 years and 32.9 kg/m^2^	7% with 0.5 mg and 9% with 0.35 mg	Semaglutide is used to manage low blood sugar levels in patients with T2DM and 56 weeks	None of the patients experienced weight gain.
Wharton et al. [24]	Patients with obesity	>18 years and >27 kg/m^2^	The mean body weight reduction varied between 7% and 14%.	2.4 mg and 68 weeks	Semaglutide at 2.4 mg caused more frequent digestive side effects than the placebo did, although they were typically mild-to-moderate and short-lived. The weight loss induced by semaglutide is largely independent of these gastrointestinal reactions.
Pratley et al. [25]	Patients with T2DM and obesity	55 years and 33.1 kg/m^2^	4.6 kg with 0.5 mg and 6.5 kg with 1 mg	0.5, 0.75, 1, 1.5 mg and 40 weeks	Semaglutide outperformed dulaglutide in enhancing blood sugar control and reducing weight in patients with T2DM.
O'Neil et al. [26]	Patients with obesity	47 years and 39.3 kg/m^2^	Loss of 13.8%	6·0% (0.05 mg), 8·6% (0·1 mg), 11·6% (0·2 mg), 11·2% (0·3 mg), 13·8% (0·4 mg), and 52 weeks	Semaglutide exhibited good tolerability over 52 weeks and achieved statistically significant weight reduction compared to placebo at all dose levels, considering diet and physical activity outcomes.
Ahrén et al. [27]	Patients with T2DM and obesity	53.7 years and >32.2 kg/m^2^	3.2–6 kg	0.5–1 mg and 56 weeks	Patients experiencing nausea or vomiting demonstrated significantly greater weight loss than those without these side effects. Weight loss ranged from -3.2 to -4.1 kg for patients on semaglutide 0.5 mg without these symptoms, whereas those on semaglutide 1.0 mg without adverse effects lost between 4.3 and 6.0 kg.
Jendle et al. [28]	Patients with T2DM and obesity	55.1 years and 32.2–33.8 kg/ m^2^	Loss of >5%	1.0 mg and 56 weeks	In the SUSTAIN 2-5 study, semaglutide treatment typically exceeded the duration of control or placebo for both weight loss categories, with most comparisons between the semaglutide 1.0 mg and control groups exhibiting statistical significance.

This review highlights the effectiveness of semaglutide in inducing weight loss among people aged 46-65 years, a group susceptible to obesity and T2DM with a mean BMI above 30 kg/m². Studies have shown that semaglutide produces significant weight reductions compared to placebo, with outcomes varying based on dosage, treatment length, and subject characteristics.

Higher doses and longer treatment periods resulted in substantial decreases in weight. Wilding et al.'s 68-week study showed semaglutide therapy resulted in an average weight reduction of -14.9%, compared to -2.4% in the placebo group (treatment difference: -12.4 percentage points; 95% CI: -13.4 to -11.5; P < 0.001) [20]. Kushner et al. demonstrated considerable reductions in weight and waist circumference with weekly 2.4 mg injections [21].

The effect of semaglutide on glycemic control was significant. Devries et al. found that semaglutide outperformed placebo and other comparators, achieving superior glycemic control without weight gain. 47-66% of patients on 0.5 mg semaglutide and 57-74% on 1.0 mg achieved HbA1c levels below 7.0%, significantly higher than placebo and comparator groups (P < 0.0001) [23].

Consistent weight loss was noted across the racial and demographic groups. DeSouza et al. reported weight reductions of 2.3-4.7 kg for 0.5 mg and 3.6-6.1 kg for 1.0 mg, with HbA1c improvements (1.0-2.0 percentage points) [22]. Wharton et al. observed dose-dependent weight loss of 7-14% over 56 weeks, with temporary mild-to-moderate gastrointestinal side effects [24].

Semaglutide surpassed other GLP-1 receptor agonists in direct comparison. Pratley et al. found semaglutide (0.5 mg and 1.0 mg) led to greater weight reductions than dulaglutide, with differences of -2.26 kg (95% CI: -3.02 to -1.51) and -3.55 kg (95% CI: -4.32 to -2.78), respectively (P < 0.0001) [25].

The association between lifestyle changes and semaglutide treatment significantly determined weight reduction results. O'Neil et al. demonstrated that combining semaglutide with a low-carbohydrate diet led to a 13.8% decrease in weight over 52 weeks [26]. However, patients reported digestive issues such as vomiting, potentially affecting treatment adherence. Ahrén et al. noted that patients experiencing nausea or vomiting during treatment tended to lose more weight [27]. Those without side effects still saw notable weight loss, ranging from 3.2-4.1 kg with the 0.5 mg dose and 4.3-6.0 kg with the 1.0 mg dose over 56 weeks.

Age-based analyses provided further insights into the effectiveness of semaglutide. Jendle et al. found that patients aged 51 years and older consistently achieved weight reduction of more than 5% when given 1.0 mg of semaglutide over 56 weeks [28]. These results align with the broader findings from the SUSTAIN 2-5 trials, which showed that semaglutide outperformed both placebo and active comparators, resulting in significant long-lasting weight loss across various patient groups.

**Figure 2:**
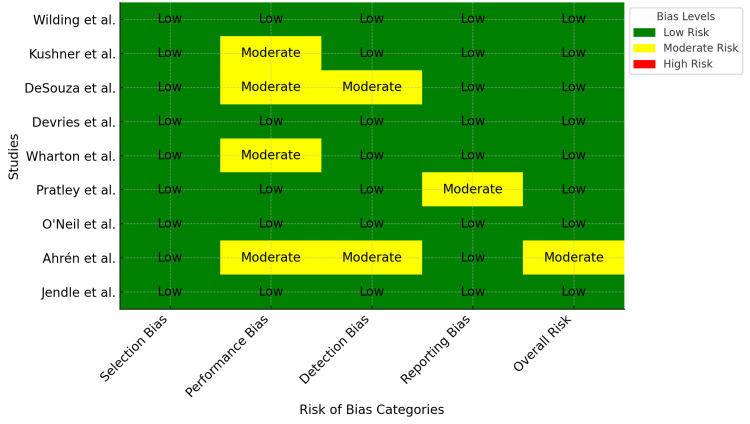
Risk of bias assessment for systematic review studies evaluating semaglutide in obesity management

The bias risk assessment among the reviewed studies varied, with the majority demonstrating a low risk across domains. All studies showed effective randomization and allocation concealment, indicating a low selection bias. Four studies exhibited moderate performance bias risk, likely resulting from intervention deviations and inadequate blinding [21,22,24,27]. DeSouza et al. and Ahrén et al. presented a moderate detection bias risk attributed to insufficient blinding of the outcome assessors [22,27]. Pratley et al. showed a moderate risk of reporting bias, potentially resulting from selective outcome reporting [25]. Except for Ahrén et al., which had a moderate overall risk owing to performance and detection biases, all studies were rated as having a low overall risk [27]. A heatmap visually summarized the findings, highlighting performance and detection bias concerns in some studies, whereas most domains consistently showed a low risk (Figure 2).

Discussion

Lingvay et al. demonstrated that semaglutide enhanced glucose absorption and reduced endoplasmic reticulum stress by downregulating genes linked to growth arrest and DNA damage [29]. This is a significant finding, as stress can induce brown adipose tissue formation [30] independently of weight loss.

Studies have consistently demonstrated that weight loss is proportional to the dosage, ranging from 3 to 15 kg. The most significant reductions were seen with higher doses, such as 2.4 mg weekly, with Wilding et al. reporting an average loss of -14.9% over 68 weeks [20]. Semaglutide was effective across various racial and ethnic groups, with consistent weight and HbA1c reduction, as noted by DeSouza et al., highlighting its adaptability in treating obesity in diverse populations [22]. Wharton et al. observed that semaglutide did not cause notable weight changes in patients with hypoglycemia under specific experimental and observational conditions, indicating its potential to stabilize weight in patients prone to rapid weight gain [24].

The dosing schedule is crucial for the weight reduction efficacy of semaglutide. Weekly administration with diet and exercise significantly reduced weight at all doses compared to that in the placebo control group over 1 year [31,32]. Wilding et al. reported a 15.3 kg weight loss in the treatment group vs. a 2.6 kg loss in the placebo group over 68 weeks [20].

A 104-week study demonstrated that semaglutide treatment resulted in greater weight loss compared to the placebo control group in patients with T2DM and obesity, with mean body weight reductions of 2.9 and 4.3 kg in the 0.5 and 1.0 mg semaglutide cohorts, respectively [32].

Combining semaglutide (0.5 mg) with metformin aids in weight control and improves HbA1c levels in patients with diabetes and obesity [29]. In a 56-week follow-up study of patients with T2DM and obesity (mean age, -54.8 years), weekly semaglutide doses of 0.5 and 1 mg led to average weight decreases of 4.28 and 6.13 kg, respectively. Compared to other GLP-1 receptor agonists, semaglutide outperformed dulaglutide and exenatide in weight loss and glycemic control, as shown by Devries et al. and Pratley et al., indicating its potential as a preferred choice for obesity management, especially in patients with T2DM [23,25].

Animal studies have demonstrated that semaglutide promotes weight loss and enhances tissue glucose uptake [33]. Patients with obesity maintained their weight when semaglutide was administered with a high-calorie diet. In patients with obesity and T2DM (aged 55 years) treated for 56 weeks, semaglutide administration at various doses resulted in significant weight loss ranging from 2.35 to 4.72 kg for 0.5 mg and 2.96 to 6.76 kg for 1.0 mg compared to the controls [31].

Although semaglutide exhibits numerous benefits, its use remains challenging. The most common adverse effect was gastrointestinal pain, which affects patient compliance [34,35]. Additionally, its high cost may limit accessibility [34]. However, the potential of semaglutide extends beyond weight control, as it exhibits promise for reducing cardiovascular risk along with providing hepatoprotective and neuroprotective benefits [8,36].

Semaglutide was generally well tolerated, although gastrointestinal side effects such as nausea and vomiting were common and dose-related, as observed by Wharton et al. and Ahrén et al. [24,27]. These effects were usually mild-to-moderate and did not significantly affect adherence or effectiveness. This review suggests that semaglutide could be a valuable addition to obesity management, particularly when combined with lifestyle changes. Its substantial weight and glycemic control effectiveness make it promising for individuals who are unresponsive to conventional treatments.

Semaglutide represents a significant advancement in obese pharmacotherapy, improving comorbid conditions such as T2DM and potentially cardiovascular health [34,36]. Its long-term efficacy and safety render it a viable option for sustained weight management [34]. Future studies should further elucidate its role in addressing other obesity-related disorders and refine its clinical applications [37].

Although semaglutide has shown efficacy in promoting weight loss, recent research indicates that tirzepatide, acting on both gastric inhibitory polypeptide and GLP-1 receptors, may be more effective. Studies have found that tirzepatide can lead to weight reductions of 7.0-9.5 kg, depending on the dose, potentially surpassing semaglutide [38,39]. Further investigations are needed to fully assess the side effects and long-term safety of tirzepatide. Comparative clinical trials are essential to determine the relative benefits of tirzepatide over other weight loss medications.

The evaluation of bias risk revealed that most studies were generally credible, with low-risk ratings in key areas. However, some studies showed moderate risks in performance and detection biases, potentially weakening their conclusions. In four studies, performance bias might have resulted from challenges in maintaining blindness during trials involving lifestyle or drug interventions [21,22,24,27]. The detection bias in the two studies suggests possible subjective measurement errors owing to assessors’ knowledge of the treatment assignments [22,27]. The study by Pratley et al. had reporting bias, highlighting the need for thorough documentation of all predetermined outcomes [25]. The consistently low selection bias across all studies indicated a strong approach to randomization and allocation concealment, reducing confounding factors, and enhancing internal validity.

Despite the proven efficacy of semaglutide for weight loss and glycemic control, certain research limitations must be acknowledged. Diversity in study designs, subject populations, and treatment compliance introduces bias that could affect real-world outcomes. Although the benefits of semaglutide are well documented, newer medications, such as tirzepatide, have shown potentially greater weight reduction effects, highlighting the need for direct comparative studies. Factors such as lifestyle differences, diet adherence, and concomitant medications have not been consistently reported across studies, requiring careful interpretation of the results. Future investigations should focus on long-term comparative trials and real-world evidence to determine the most effective obesity management strategy.

## Conclusions

Semaglutide has demonstrated remarkable effectiveness in reducing weight and controlling blood sugar levels, making it a promising drug for treating obesity. Research indicates that weekly administration of semaglutide results in substantial weight loss, with average reductions of up to 14.9% over a 68-week period at a 2.4 mg dosage. The observed improvements in HbA1c levels further underscore its potential in addressing obesity-related metabolic issues. Despite its clinical advantages, the widespread use of semaglutide is hindered by its high cost, and gastrointestinal side effects may affect long-term patient compliance. While its short-term safety is well established, additional research is needed to assess long-term risks, particularly concerning heart health outcomes and sustained weight management.

Although semaglutide has demonstrated significant weight loss and glycemic control benefits, several limitations should be considered. Variability in trial design, patient demographics, and treatment adherence introduces potential biases that may impact real-world effectiveness. Furthermore, while the benefits of semaglutide are well established, emerging therapies such as tirzepatide have shown potentially superior weight loss effects, highlighting the need for direct comparative trials. Additionally, confounding factors such as lifestyle variations, dietary adherence, and concurrent medications have been inconsistently reported across studies, necessitating cautious interpretation of the findings.

Future studies should focus on extended comparative analyses of semaglutide and emerging obesity treatments to determine their relative effectiveness, safety, and patient adherence trends. Real-world data from diverse populations are essential for evaluating its performance in controlled trial environments. Additionally, prioritizing cost-effectiveness studies is crucial for guiding healthcare policy decisions regarding accessibility and long-term affordability.
